# Xenon and Sevoflurane Provide Analgesia during Labor and Fetal Brain Protection in a Perinatal Rat Model of Hypoxia-Ischemia

**DOI:** 10.1371/journal.pone.0037020

**Published:** 2012-05-17

**Authors:** Ting Yang, Lei Zhuang, António M. Rei Fidalgo, Evgenia Petrides, Niccolo Terrando, Xinmin Wu, Robert D. Sanders, Nicola J. Robertson, Mark R. Johnson, Mervyn Maze, Daqing Ma

**Affiliations:** 1 Anesthetics, Pain Medicine and Intensive Care, Department of Surgery and Cancer, Imperial College London, Chelsea and Westminster Hospital, London, United Kingdom; 2 Department of Anesthesia and Intensive Care, Beijing University, The First Hospital, Beijing, China; 3 Department of Anesthesia and Perioperative Care, University of California San Francisco, San Francisco, California, United States of America; 4 Neonatology, Institute for Women's Health, University College London, London, United Kingdom; 5 Reproductive Biology, Department of Surgery and Cancer, Imperial College London, Chelsea and Westminster Hospital, London, United Kingdom; University of Washington, United States of America

## Abstract

It is not possible to identify all pregnancies at risk of neonatal hypoxic-ischemic encephalopathy (HIE). Many women use some form of analgesia during childbirth and some anesthetic agents have been shown to be neuroprotective when used as analgesics at subanesthetic concentrations. In this study we sought to understand the effects of two anesthetic agents with presumptive analgesic activity and known preconditioning-neuroprotective properties (sevoflurane or xenon), in reducing hypoxia-induced brain damage in a model of intrauterine perinatal asphyxia. The analgesic and neuroprotective effects at subanesthetic levels of sevoflurane (0.35%) or xenon (35%) were tested in a rat model of intrauterine perinatal asphyxia. Analgesic effects were measured by assessing maternal behavior and spinal cord dorsal horn neuronal activation using c-Fos. In separate experiments, intrauterine fetal asphyxia was induced four hours after gas exposure; on post-insult day 3 apoptotic cell death was measured by caspase-3 immunostaining in hippocampal neurons and correlated with the number of viable neurons on postnatal day (PND) 7. A separate cohort of pups was nurtured by a surrogate mother for 50 days when cognitive testing with Morris water maze was performed. Both anesthetic agents provided analgesia as reflected by a reduction in the number of stretching movements and decreased c-Fos expression in the dorsal horn of the spinal cord. Both agents also reduced the number of caspase-3 positive (apoptotic) neurons and increased cell viability in the hippocampus at PND7. These acute histological changes were mirrored by improved cognitive function measured remotely after birth on PND 50 compared to control group. Subanesthetic doses of sevoflurane or xenon provided both analgesia and neuroprotection in this model of intrauterine perinatal asphyxia. These data suggest that anesthetic agents with neuroprotective properties may be effective in preventing HIE and should be tested in clinical trials in the future.

## Introduction

Neonatal hypoxic ischemic encephalopathy (HIE) affects 1–3 per 1000 term births in the developed world [1], associating with high mortality and lifelong chronic disabilities [2], [3]. Ten to 15% of infants with moderate to severe neonatal encephalopathy will die; 15% of the survivors will develop cerebral palsy and a larger proportion will present with significant disabilities including global developmental delay, cognitive problems, deafness and epilepsy. Although therapeutic hypothermia has been proven effective and safe in reducing neurological deficits and mortality [4], a large proportion (up to 50%) of treated infants will encounter adverse outcomes. On a global scale perinatal asphyxia is an immense problem with almost one quarter of the world's 4 million neonatal deaths being attributed to perinatal asphyxia [5]. Thus recognizing, treating, and preventing intra-uterine hypoxia-ischemia is one of the main priorities of perinatal medicine.

We previously reported on the neuroprotective effects of xenon and sevoflurane preconditioning in a model of neonatal asphyxia [6]. The *in vivo* study assessed 7 day old rats but could not effectively reproduce the intra-uterine environment in which HIE normally occurs. In the present study we have used a validated model of nociceptive parturition [7] and intrauterine ischemic brain damage [8] to test the ability of xenon and sevoflurane in providing labor analgesia and neuroprotection.

## Materials and Methods

The study was approved by the Home Office (UK) and conforms to the United Kingdom Animals (Scientific Procedures) Act of 1986. All efforts were made to minimize animal suffering and the number of animals used. Pregnant Sprague-Dawley (SD) rats (300–350 g) were housed in individual cages in a 12-h-light/12-h-dark cycle in temperature (22–23°C) and humidity-controlled environment with free access to standard laboratory chow.

### Labor analgesia

We used a validated parturition nociceptive model [7] to investigate the analgesic efficacy of xenon and sevoflurane. On gestation day 21, SD rat dams received 5 mg/kg Mifepristone (Sigma, UK) by oral gavage to induce parturition the following day [7]. On gestation day 22, the laboring rats were placed in a transparent anesthetic chamber with a video camera positioned below the chamber to record the behavior of the dams from 9 am until the first pup was delivered. Dams received 0.35% sevoflurane or 35% xenon (Air Liquide, UK) in 30% oxygen balanced by nitrogen. Both concentrations were obtained from our pilot studies, in which we tested sevoflurane or xenon from high concentrations (0.8% and 50% respectively) to lower levels gradually. We found 0.35% sevoflurane or 35% xenon did not affect respiratory function by counting respiratory rate but not blood gas measurement due to technical difficulty and not prolong the duration of labor. Animals from naïve or control group received 30% oxygen plus 70% nitrogen.

**Behavioral analysis:** The numbers of stretching behaviors, either inward turning of one hind paw or straining and squashing of the lower abdomen to the floor [7], were counted offline by an investigator blinded to the experimental protocol for the 90 min period preceding the expulsion of the first pup.

**Immunohistochemistry:** One hour after giving birth to the first pup, the dams were deeply anesthetized with sodium pentobarbital (100 mg/kg, i.p.) and perfused transcardially with ice-cold heparinized 0.1 M phosphate buffer solution (PBS) followed by 4% paraformaldehyde (PFA) in 0.1 M PBS at pH 7.4 (VWR International, UK), the whole spinal cord was removed. After 24 hours fixation in 4% PFA, the lumbosacral enlargement of the spinal cord was embedded in optimal cutting temperature compound (OCT, Sakura, UK) and sectioned transversely at 30 µm intervals and stained for c-Fos as previously described [9]. Briefly, sections were washed in 0.01 M PBS, quenched for 30 min in a solution of methanol containing 3% hydrogen peroxide, then incubated for 1 h in blocking solution (3% normal donkey serum), followed by incubation overnight in rabbit anti c-Fos antibody (1∶500, Santa Cruz, UK). Following incubation with primary antibody, the sections were incubated for 1 h in secondary antibody (1∶200 donkey anti-rabbit, Millipore, UK). Afterwards, ABC reagent was applied (standard Vectastain ABC Elite Kit, Vector Labs, UK) for 2 h. Staining was revealed by 3,3′-diaminobenzidine (DAB, Vector Labs, UK) for 5 min for positive c-Fos cell quantification analysis. Counting of cells was made by an investigator blinded to the experimental protocol by 40× magnification. Four high magnifications in the dorsal horn layer I–II at the lumbosacral of spinal cord were chosen randomly from each slide, and three slides were evaluated of each animal.

### Fetal asphyxia

Separate cohorts of dams (without mifepristone treatment) received 0.35% sevoflurane or 35% xenon in 30% oxygen balanced by nitrogen for 4 hours on gestation day 22 from 8am. Animals from naïve or control group received 30% oxygen plus 70% nitrogen. Dams were sacrificed by cervical dislocation and uterine horns were removed and placed in a water-bath at 37°C for 10 min to induce hypoxic-ischemic insult 4 hours after gas exposure. The dams giving birth before the surgery and the pups they delivered were discarded from the study. Fetal asphyxia was performed as previously described [8]. Pups were rapidly delivered from the uterus, the umbilical cord ligated, and pups were cleaned and manually stimulated to initiate breathing in an incubator at 37°C in air, for 1 hour. Pups born after a spontaneous vaginal delivery were used as the naïve control group. After cesarean delivery, pups were placed with a surrogate mother for subsequent nurturing. Pups were allowed to live for 3, 7 or 50 days [8].

**Immunohistochemistry and histology:** On Postnatal Day (PND) 3 and 7 [8], pups were deeply anesthetized with pentobarbital (100 mg/kg, i.p.) and perfused with 4% PFA. Brains were harvested and stained for caspase-3 (1∶5000, Cell Signaling Technology, UK) as previously described [8]. Paraffin sections, 5 µm in thickness, were stained with cresyl violet. Staining was obtained by 0.5% cresyl violet solution in distilled water and the slides were de-colored with 95% ethanol. Photomicrographs were taken using an Olympus BX-60 microscope and captured with a Zeiss KS-300 color 3CCD camera (three images/animals in CA1 area). The counting for caspase-3 positive cells or cresyl violet staining healthy cells was performed by an investigator who was blinded to the interventional group. In our previous work, we demonstrated that after the intrauterine ischemia/hypoxia injury, the differences of neuroapoptosis and neuron death between the injury groups and control groups are consistent on PND 1, 3 and 7. Because of the excessive neuronal sprouting and neurogenesis on PND 1 and 3, we use PND 7 as the time point for evaluation of morphological change in hippocampus. However, PND 3 is preferable for caspase 3 evaluation because this enzyme is rapidly degraded and hard to be assessed at PND 7 [8].

**Neurocognitive Testing with Morris Water Maze (WM):** Neurocognitive outcome was evaluated daily (starting on PND 50) using Morris water maze (WM) [10] with a computerized video tracking system (EthoVision®; Noldus, Wageningen, The Netherlands). Briefly, WM consisted of a circular pool 110 cm in diameter and 60 cm in height, filled to a depth of 30 cm with water (24±0.5°C). A hidden submerged platform was placed in one quadrant 2.5 cm below water surface. Rats were placed in the water in a dimly lit room with visual clues around room wall. The time of locating the submerged platform (defined as the latency-cut off time 90 sec) was measured. The longer the time required to locate the platform indicates the more impairment in spatial learning and memory components of neurocognition. Four trials tests were performed for each rat every day. Each trial started from a different quadrant. Testing was consecutively repeated for 5 days. On testing day 6, each animal was subjected to a probe trial (60 s cut off), where they were tested in the absence of the platform. The time spent in the 20-cm-diameter area where the platform was located during previous testing was recorded and represents an index of memory.

### Data analysis

The results are expressed as mean ± SEM. Data was analyzed with one-way analyses of variance followed by *post hoc* Newman-Keuls test wherever appropriate or unpaired t-test using SPSSv13.0 (SPSS lnc. Chicago, IL, USA). A p value<0.05 was considered to be a statistically significant.

## Results

### Labor analgesia

**Figure 1:**
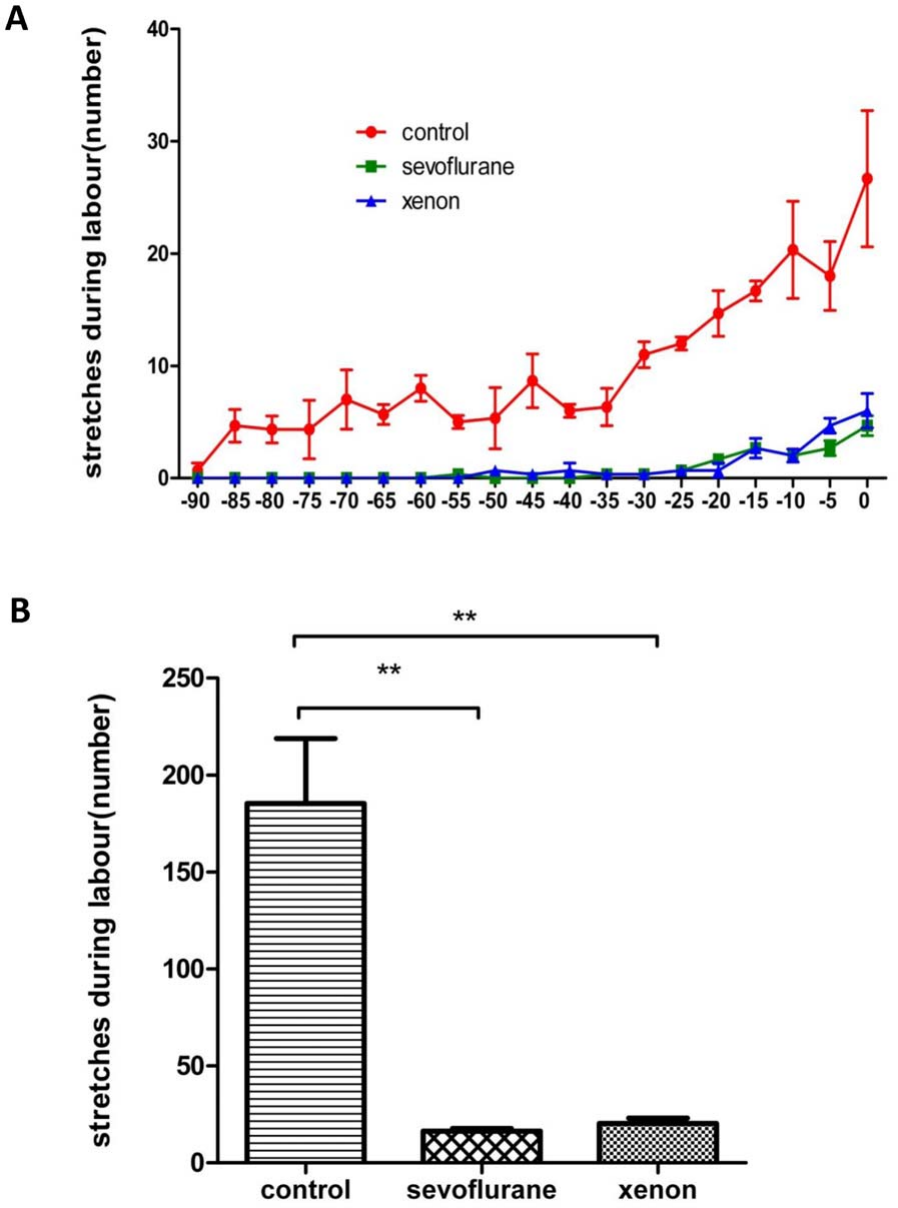
Pain stretching behavior during labor. The time course (**A**) and counting (**B**) of stretches, either inward turning of the hind paw or straining of the abdomen, which represent uterus contraction-related pain 90 min prior to birth delivery. Dams from sevoflurane or xenon groups had a significant decrease of stretches compared to control group. Data are expressed as mean ± SEM (n = 4); **p<0.01. Bar = 100 µm.

**Nociceptive behavior-labor pain:** In order to assess the effects of anesthetic exposure on pain-related behavior we measured stretches. Following exposure to either xenon or sevoflurane, uterine contraction-related pain (stretching behavior) in the 90 min period prior delivery was lower in the xenon (46±6) and sevoflurane (31±2) groups as compared to controls (oxygen enriched air exposure only) (185±47) prior to delivery of the first pup (p<0.01, n = 4) (Figure 1).

**Figure 2:**
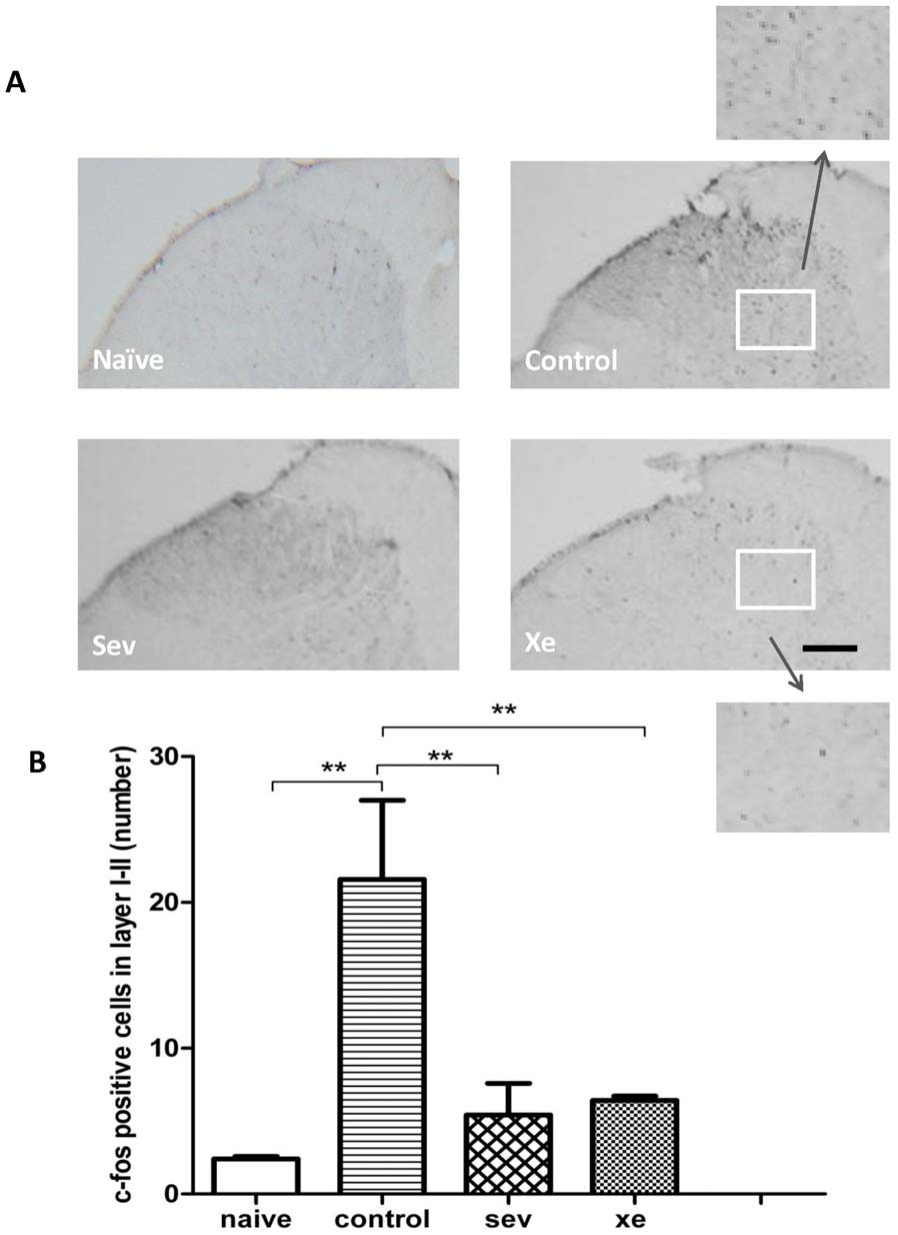
c-Fos expression in the spinal. Rats were divided into naïve group (virgin female rats exposed in 30% oxygen balanced by 70% nitrogen), control group (parturient rats exposed in 30% oxygen balanced by 70% nitrogen), sevoflurane group (parturient rats exposed in 0.35% sevoflurane in 30% oxygen balanced by nitrogen), or xenon group (parturient rats exposed in 35% xenon in 30% oxygen balanced by nitrogen). Photomicrographs show c-Fos expression in the dorsal horn at the lumbosacral level of the spinal cord (**A**). **B** shows the quantification of c-Fos positive cells. Data are expressed as mean ± SEM (n = 4). **p<0.01. Bar = 100 µm. The inserted box indicates the area being subjected to high magnification image.

**Neuronal activation during labor pain:** Next we measured the effects of xenon or sevoflurane as labor analgesics on neuronal activity using c-Fos expression in the spinal cord. One hour after delivery of the first pup, the number of c-Fos positive neurons in the spinal cord layer I–II was significantly higher in the control group (22±5) as compared to those in the naïve group (2±0.2), xenon group (5±2) and sevoflurane group (6±0.3, p<0.01, n = 4, Figure 2), indicating that xenon or sevoflurane at the administered concentration can prevent the nociception induced by labor in rats.

### Fetal asphyxia

**Figure 3:**
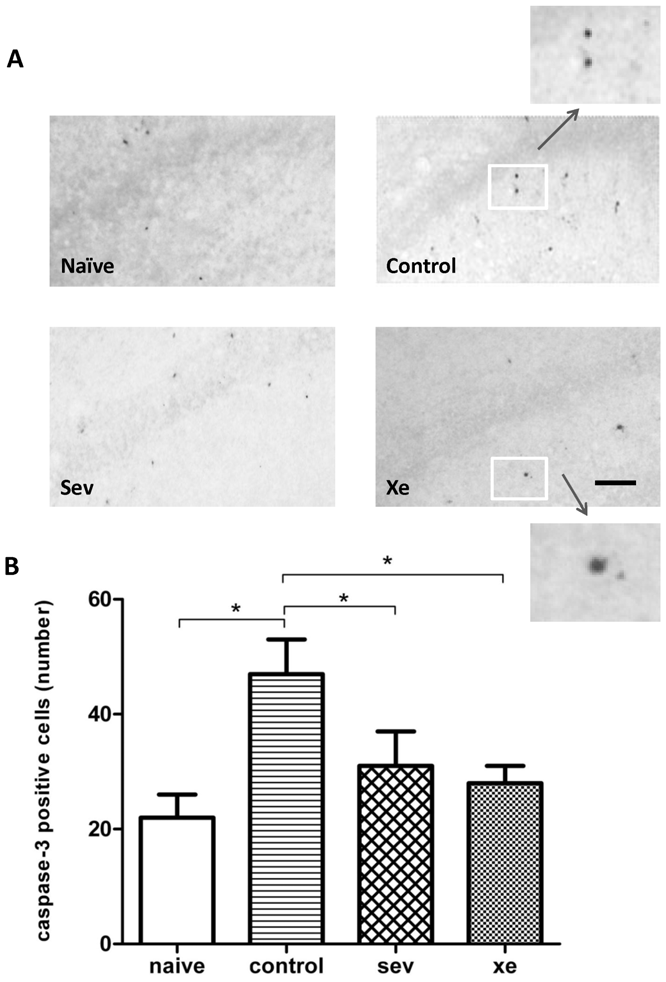
Caspase 3 expression in the hippocampus. Pups from control, sevoflurane or xenon groups experienced intrauterine hypoxia, when the dams were exposed in different gases during labor. Pups from naïve group were from vaginal delivery from surrogate mother. Photomicrographs show caspase 3 expression in the hippocampus on PND3, CA1, (**A**, 20×). **B** shows the quantification of caspase 3 positive cells. Results are expressed as mean ± SEM (n = 5). *p<0.05. Bar = 100 µm. The inserted box indicates the area being subjected to high magnification image.

**Figure 4:**
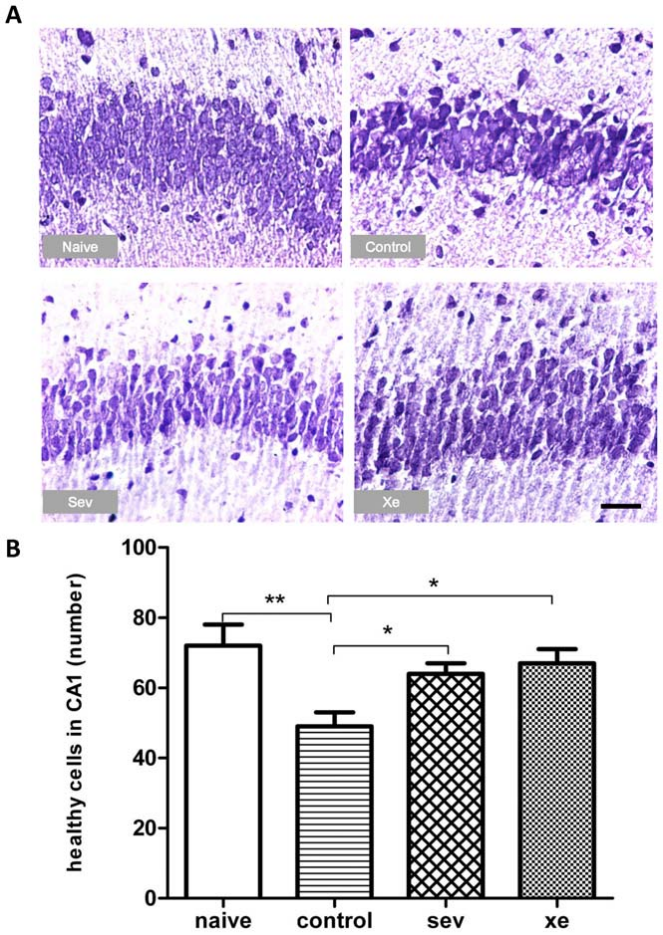
Morphological changes in the hippocampus. Photomicrographs show cresyl violet staining in CA1 region of hippocampus (**A**, 40×) on PND 7. Neuronal loss, cellular disorganization and shrinkage were attenuated in sevoflurane or xenon groups compared to control group. There is a significant difference in the number of healthy cells in control group, compared to all other groups (**B**). Results are mean ± SEM (n = 6). * p<0.05, **p<0.01.

**Asphyxia causes apoptosis, which is ameliorated by xenon and sevoflurane:** To assess the protective effects of xenon or sevoflurane exposure prior to injury on the brain, the neuronal cell injury or cell death were measured by caspase-3 and cresyl violent staining on PND 3 and 7 respectively. An example of a high magnification area from the hippocampus (CA1) is illustrated in Figure 3A. The pups from the dams preconditioned by sevoflurane or xenon had significantly less caspase-3 positive cells (31±6, 28±3 respectively) after asphyxia when compared to the pups exposed to hypoxia alone (47±6, p<0.05, n = 6) at PND 3. The number of caspase-3 positive cells in the naïve controls (vaginal delivery) was 22±4, which is significantly lower than pups exposed to hypoxia alone (p<0.05, n = 6), but overall not different from sevoflurane or xenon (Figure 3B). To assess morphological changes in the brain and to corroborate the caspase-3 findings, we used sections stained with cresyl violet to assess neuronal loss. Minor changes were observed in cortical areas, however the hippocampus showed most robust and significant differences, with clear neuronal loss in the control group as compared to any other treatment on PND 7 (p<0.01, Figure 4).

**Figure 5:**
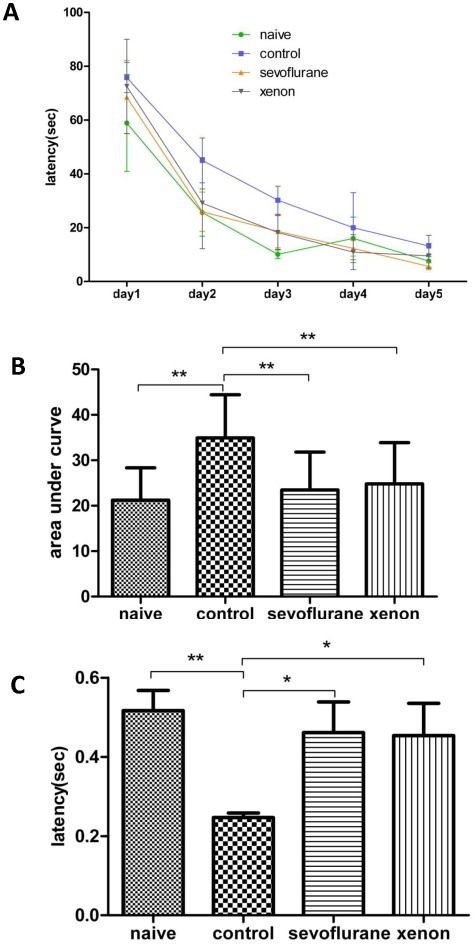
Neurocognitive behavior at PND 50. **A** shows the average latencies, the time required for each animal to reach the hidden platform, of the four trials for the initial 5 days. **B** shows the area under curve (AUC) derived from **A**. **C** shows the percentage of the time the rats spent in the platform area in the probe trial on the 6^th^ testing day. Control group have significant neurocognitive impairment when compared to naïve group. There is no difference between sevoflurane, xenon groups and naïve group. **D** shows there is no difference in the average velocity in each group. Data are expressed as mean ± SEM (n = 5–6) and compared by one-way analysis of variance and Student-Newman-Keuls method. * p<0.05, **p<0.01.

**Postnatal memory deficit is improved following treatment with xenon and sevoflurane:** In order to assess neuropathological amelioration afforded by xenon or sevofluane exposure prior to injury leading to cognitive improvement, we used WM to assess learning and memory functions at the juvenile stage on PND 50. Latency to locate the hidden platform was significantly decreased in animals exposed to sevoflurane or xenon as compared to pups exposed to hypoxia alone (Figure 5A). We used the Area under Curve (AUC) to denote the cumulative average time of the four trials based on the five testing days. There was a significant difference comparing pups exposed to hypoxia alone with naïve group (p<0.05, Figure 5A and B). Pups exposed to hypoxia alone spent a shorter time in the platform area in the probe trial as compared to naïve, sevoflurane or xenon treated groups (p<0.05, Figure 5C). There was no significant difference in swimming speed among groups (Figure 5D).

## Discussion

In this study we have demonstrated that (i) peri-labor administration of the anesthetic agents sevoflurane or xenon provide effective alleviation of nociception induced by labor in rats, and (ii) preconditioning with either agent for four hours prior to fetal asphyxia offered neuroprotection and ameliorated cognitive dysfunction.

The visceral component of labor pain, from the uterus to the lower thoracic and upper lumbar spinal cord segments, relays on c-fiber afferent signaling [11]. C-Fos is a prototypical neuronal marker for nociceptive signaling in spinal cord [12]. Because of the cross-innervations of nerves in the spinal cord, c-Fos expression as induced by parturition is detectable through segments T12-S2 of the spinal cord, but in particular between L5-S1. Immunohistochemistry study demonstrated that c-Fos expression is detectable in the spinal cord 30 min after peripheral nociceptive stimulation, with levels peaking between 60 and 90 min [12]. Concurrent to these molecular changes, stereotypic behavior associated with the visceral nociception in response to uterine stimulation can be assessed in rats [7], [13]. In our current study, we measured nociceptive behavior assessing stretches and c-Fos expression in the spinal cord 1 h after delivery of the first pup. The number of uterine contraction-related stretches significantly increased with time until all pups were delivered (Figure 1B). This is consistent with the clinical observations that labor pain increases with time and peaks during the second stage of labor and during delivery of the baby. Here we demonstrated that the administration of xenon or sevoflurane to the dam remarkably reduced dam's stretches before delivery (Figure 1A). This was associated with significantly lower c-Fos expression in the dorsal horn of the spinal cord as compared to untreated control (Figure 2). The changes in behavior and c-Fos expression following xenon or sevoflurane administration are similar to the effects of epidural analgesia with morphine [7], suggesting that both 35% xenon and 0.35% sevoflurane exert antinociceptive effect during labor in rats. A clinical trial showed the best concentration of sevoflurane for labor analgesia was 0.8% and, though sedation was noticed, it was safe and associated with higher maternal satisfaction than nitrous oxide [14]. In our pilot studies we saw severe sedation of the dams and delayed labor with sevoflurane concentrations >0.5%. This may be due to the different administration method: in the clinical trial patients used sevoflurane intermittently according to their needs, whereas in our animal model, sevoflurane is given continuously. However we cannot exclude that the behavioral changes we observed were not due to increased sedation rather than antinociception which has been well documented previously in this model with morphine administrated intrathecally [13], hence the importance of the c-Fos data to confirm antinociceptive effects.

The analgesic effects of xenon and sevoflurane have been proven pre-clinically and clinically [14], [15], [16], [17], [18], [19]. Xenon appears to exert its antinociceptive effect, at least partially, by directly acting on the spinal cord [20], conversely nitrous oxide has limited effects on the spinal cord [21]. It has been suggested that the analgesic properties of sevoflurane may be mediated by GABAergic signaling [22], however the specific molecular mechanisms of the analgesic effects of inhalational aesthetics remain unclear.

Our previous studies demonstrated that xenon and sevoflurane protected the neonatal neuronal system from brain injury [6], suggesting that xenon and sevoflurane may be ideal alternatives to nitrous oxide for labor analgesia, with the added benefit of protecting the neonates from the unanticipated hypoxic-ischemic injury. To investigate whether xenon or sevoflurane could serve this dual function during labor, we used our established model of perinatal asphyxia [8], in which the hypoxic insult occurs in utero during labor and can lead to global brain injury. This model closely simulates the course of perinatal ischemia and hypoxia and is consequently more suitable for testing potential therapeutic approaches to protect the vulnerable fetal brain from the consequences of ischemia and hypoxia. This study shows that the administration of 35% xenon or 0.35% sevoflurane to pregnant dams reduced neuronal apoptosis and cell death in the hippocampus of the pups (Figure 3 & 4). This was associated with attenuation in long-term cognitive dysfunction following intrauterine ischemia/hypoxia injury (Figure 5).

The neuroprotective properties of xenon and sevoflurane has been evaluated in both *in vivo* and *in vitro* studies [6], [9], [23], [24], [25], [26], [27], [28], [29], but the underlying mechanisms have not been clearly identified. Xenon is a competitive *N*-methyl-D-aspartate (NMDA) receptor antagonist at the glycine site [30], [31], which may contribute to xenon-induced neuroprotective effects [32]. *In vitro* work showed that the adenosine triphosphate-sensitive potassium (K_ATP_) channel can also be activated by xenon [33], however sevoflurane preconditioning possibly relies on mitochondrial K_ATP_ [34]. In our recent study, we demonstrated that xenon alone or in combination with sevoflurane protects against ischemic injury and the effect is mediated by PI3K and pCREB signaling pathway [6], [25], [35]. In addition, sevoflurane is thought to provide neuroprotection via anti-inflammatory effects which is a possible mechanism of protection against perinatal brain injury [36], [37].

Xenon does not appear to be teratogenic nor has been associated with neurodegeneration and has been used in an apparently safe manner in neonates for many years for neuroimaging. However there are some reports about sevoflurane which are less reassuring although it is much less toxic than isoflurane at the equipotent concentration [38]. One study suggested after exposure to 3% sevoflurane, the mice developed learning deficit and abnormal social behavior [39]. Another acute gas exposure model showed 6 h sevoflurane exposure (2.1% or 3%) may induce neurotoxicity in 7-day-old mice especially in Alzheimer disease transgenic mice [40]. It is important to note that in these studies sevoflurane was used at concentrations of 2.1% or 3.0%. However, in the labor analgesia setting, sevoflurane is used at much lower concentration [14] hence the neonatal toxicity may be less of a concern. Due to technical difficult of the nature work, the blood gases were not monitored during labor analgesia period although the respiratory rate was within the normal physiological range. Therefore, blood gas changes induced by anesthetic exposure, if any, would be minimal and would not distort our conclusions, i.e. fetal brain protection which was assessed a few hours after anesthetic exposure.

### Conclusion

Our data indicate that both xenon and sevoflurane administered for 4 h before asphyxia at concentrations that can provide antinociception during labor and also protect against intrauterine fetal hypoxic-ischemic brain injury in an experimental rat model of hypoxia/ischemia. These data suggest that in the clinical setting, xenon or sevoflurane for labor analgesia may not only provide effective analgesia but also prevent brain injury in the compromised fetus that extends into neuroprotection of the newborn following birth.
